# Giant chronic splenic infarction mimicking splenic neoplasm in a patient with rheumatic heart disease: a case report

**DOI:** 10.3389/fcvm.2026.1850185

**Published:** 2026-07-21

**Authors:** Wei Tian, Xu Deng, Chunyuan Yang, Jianxing Tian, Zonglong Zhu, Rui Huang, Wei Pan

**Affiliations:** Department of Hepatobiliary and Pancreatic Surgery, The People’s Hospital of Lezhi, Ziyang, China

**Keywords:** misdiagnosis, rheumatic heart disease, splenectomy, splenic infarction, splenic neoplasm

## Abstract

**Background:**

Giant chronic splenic infarction is an extremely rare condition with insidious onset and atypical imaging features, carrying a high risk of misdiagnosis as splenic neoplasm. This case highlights the atypical clinical features and important diagnostic considerations of this uncommon disorder.

**Case description:**

A 73-year-old female presented with recurrent epigastric pain for 2 years, with a medical history of rheumatic heart disease and hypertension. Contrast-enhanced abdominal computed tomography showed a huge splenic mass measuring approximately 18.0 × 14.2 × 19.1 cm with heterogeneous enhancement and calcification, which was highly suggestive of splenic neoplasm. The patient received splenectomy, and pathological examination confirmed extensive splenic infarction with focal fibrosis and calcification. The patient had an uneventful postoperative recovery, with no evidence of thrombosis or infectious complications during the 3-month follow-up period.

**Conclusion:**

Rheumatic heart disease with mitral stenosis may have contributed to splenic infarction as a possible cardioembolic source in this patient. Giant chronic splenic infarction may be misdiagnosed because of atypical clinical manifestations, tumor-mimicking imaging features, and insufficient integration of clinical, laboratory, and imaging findings. A comprehensive assessment of clinical history, laboratory results, imaging findings, and cardiac status may help reduce misdiagnosis and guide appropriate management.

## Introduction

Splenic infarction occurs when branches of the splenic artery become obstructed, leading to tissue ischemia and necrosis. Typical imaging findings are well-defined, wedge-shaped peripheral lesions (1, 2). Giant chronic splenic infarction is rare and may gradually form a huge mass with organization, fibrosis, and calcification (3). Its clinical and radiologic features can closely mimic splenic neoplasm, leading to frequent misdiagnosis. We present this case to clarify the etiology, diagnostic pitfalls, and clinical lessons for optimal clinical practice.

## Case presentation

A 73-year-old female presented to The People's Hospital of Lezhi in January 2026 with recurrent epigastric pain for 2 years. The chief complaint was intermittent left upper abdominal pain, occasionally associated with nausea and minor non-bilious vomiting. There was no history of chills or fever. The patient had a history of rheumatic heart disease and hypertension, but no history of trauma or surgery. She denied regular cardiac evaluations or anticoagulant treatment.

**Figure 1:**
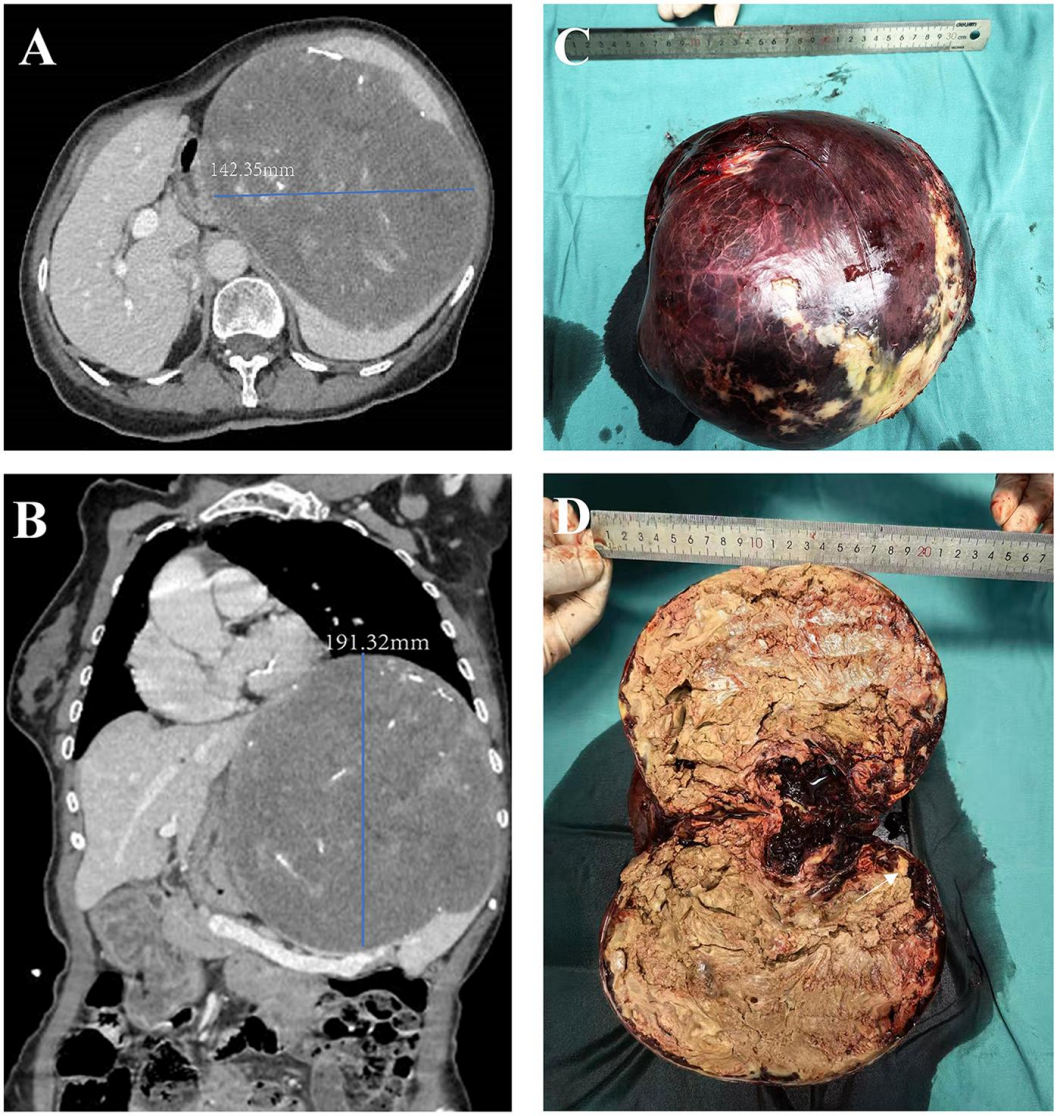
**(A)** Axial contrast-enhanced CT image in the portal venous phase showing a large heterogeneous splenic mass. **(B)** Coronal reconstruction demonstrating mass effect on adjacent organs. **(C)** Gross specimen of the spleen after splenectomy. **(D)** Cut section of the specimen showing focal calcification (white arrow).

**Figure 2:**
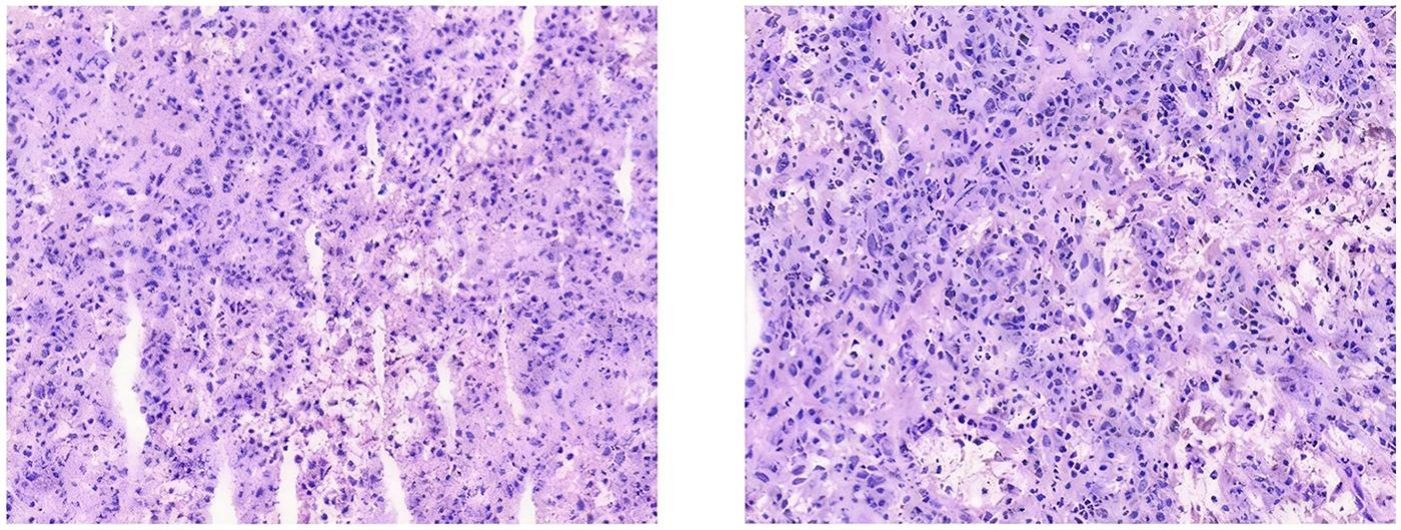
Histopathological examination showing splenic infarction with tissue necrosis and fibrosis (hematoxylin and eosin stain, original magnification × 200).

Physical examination revealed marked distension of the left upper abdomen with a palpable mass demonstrating firm consistency and limited mobility; no superficial peripheral lymphadenopathy was detected. Contrast-enhanced abdominal computed tomography (CT) revealed a large mass in the left upper abdomen measuring approximately 18.0 × 14.2 × 19.1 cm. The mass contained patchy areas with mildly increased attenuation and calcification and capsular calcification. Portions of the lesion had indistinct borders with the spleen. After contrast administration, peripheral rim enhancement and septal enhancements were observed. The spleen, diaphragm, stomach lumen, and adjacent tissues were compressed (Figures 1A,B). Cardiac enlargement and calcification were observed in the aorta and portions of the valves. Echocardiography revealed rheumatic heart disease, calcified plaques on the mitral and aortic valves, moderate mitral stenosis with mild regurgitation, as well as mild tricuspid and mild aortic regurgitation. Complete blood count showed low hemoglobin and platelet levels, with a hemoglobin level of 107 g/L (normal range: 115–150 g/L) and a platelet count of 90 × 10^9^/L (normal range: 125–350 × 10^9^/L), which might be related to hypersplenism secondary to chronic splenic infarction. The coagulation profile was within the normal range, including prothrombin time of 12.80 s, activated partial thromboplastin time of 29.50 s, international normalized ratio of 1.07, and fibrinogen level of 2.50 g/L. The D-dimer level was not available. The 12-lead electrocardiogram and tumor marker levels, including AFP, CEA, CA-199, CA-125, and CA-153, were normal. A multidisciplinary team (MDT) consultation involving hepatobiliary surgeons, medical oncologists, radiologists, and pathologists was conducted to formulate the optimal therapeutic strategy. No definite evidence of malignancy was identified on imaging studies or laboratory tests; however, surgical resection was deemed necessary due to the mass effect and diagnostic uncertainty. After obtaining informed consent from the patient and her family members, splenectomy was performed. Specimens of the splenic mass and spleen were measured (Figures 1C,D). Pathological analysis of the surgical specimen indicated an extensive splenic infarction with focal fibrosis and calcification (Figure 2). The patient had an uneventful recovery and was discharged on postoperative day 10.

At the 3-month telephone follow-up after discharge, the patient reported a good mental status, with no evidence of venous thrombosis, pulmonary infection, or abdominal infection. Given that the patient's CHA₂DS₂-VASc score was 3, indicating high thromboembolic risk, long-term oral anticoagulation was indicated.

## Discussion

Massive chronic splenic infarction shows a bimodal age distribution, with its etiology shifting from hematological disorders in younger patients to cardioembolic events in older adults (4). It may result from cardiac thromboembolism, hematological disorders, and iatrogenic factors such as partial splenic embolization (5). Notably, the misdiagnosis rate can be as high as 30%–50%, and the condition is most commonly misdiagnosed as a splenic neoplasm or abscess (6). The present case is noteworthy because a giant chronic splenic infarction was initially suspected to be a splenic neoplasm, highlighting the diagnostic difficulty of this uncommon presentation.

Rheumatic heart disease with mitral stenosis may have contributed to the development of splenic infarction in this patient as a possible cardioembolic source. The patient had a history of rheumatic heart disease, and echocardiography confirmed rheumatic valvular disease complicated by moderate mitral stenosis. This valvular lesion can impair left atrial blood flow and promote hemodynamic stasis, thereby increasing the risk of thromboembolic events (7). If thrombi form and detach, they may embolize the splenic artery or its branches, resulting in splenic ischemia and necrosis (8). However, no direct evidence of intracardiac thrombus, embolic material, or definite splenic arterial embolism was identified in this case. Therefore, the exact etiology of the splenic infarction could not be definitively established. Over the 2-year disease course, the infarcted splenic tissue may have gradually undergone organization, fibrosis, and calcification, eventually developing into a giant space-occupying lesion. This pathological process was consistent with the patient's chronic abdominal pain and giant splenic mass.

Approximately 20% of reported cases of splenic infarction are asymptomatic and may be detected incidentally, contributing to delayed diagnosis (9). The patient did not present with typical manifestations of acute splenic infarction, including sudden left upper abdominal pain, tenderness, nausea, or vomiting (10). The acute phase of splenic infarction was insidious and unrecognized; instead, the patient only presented with recurrent chronic upper abdominal pain that persisted for 2 years. This clinical presentation was highly consistent with chronic abdominal pain secondary to the mass effect of a splenic lesion, leading clinicians to initially favor neoplastic lesions in the differential diagnosis.

On contrast-enhanced CT, the lesion appeared as a giant space-occupying mass with heterogeneous enhancement, internal calcification, and marked compression of adjacent organs, rather than the typical wedge-shaped hypodense pattern of acute splenic infarction. The main differential diagnoses included primary splenic neoplasm, hemangioma, abscess, and chronic hematoma. Because calcification may also be observed in certain splenic tumors or vascular lesions (11), the radiological appearance favored a neoplastic or hemangiomatous lesion before surgery. A similar diagnostic pitfall has been reported in chronic splenic infarction misdiagnosed as splenic neoplasm (12).

Preoperatively, percutaneous splenic biopsy was not performed to confirm the pathological nature of the lesion. Although image-guided splenic biopsy can provide diagnostic information in selected cases, previous studies have reported potential bleeding-related complications (13, 14); therefore, its use should be carefully individualized according to lesion characteristics and procedural risk. In the present case, the lesion was extremely large, compressed adjacent organs, and malignancy could not be completely excluded before surgery. After multidisciplinary discussion and informed consent, splenectomy was performed because of the marked mass effect and the need for definitive diagnosis and treatment. This case illustrates a common diagnostic pitfall. When a splenic lesion is extremely large and shows heterogeneous enhancement with calcification, clinicians may initially favor a neoplastic process. However, negative tumor markers, long-standing abdominal symptoms, and potential thromboembolic risk factors should prompt consideration of chronic splenic infarction. A comprehensive assessment combining clinical history, laboratory results, imaging findings, and cardiac evaluation may help reduce diagnostic bias and avoid overreliance on radiological appearance alone.

## Conclusion

Giant chronic splenic infarction should be included in the differential diagnosis of massive splenic space-occupying lesions, particularly in patients with potential thromboembolic risk factors such as rheumatic mitral stenosis. Although the exact etiology may not always be definitively established, a comprehensive assessment integrating clinical history, cardiac evaluation, imaging features, and laboratory findings is essential to avoid misdiagnosis and guide appropriate management.

## Data Availability

The original contributions presented in the study are included in the article/Supplementary Material, further inquiries can be directed to the corresponding author.
